# Thermal control of the defunctionalization of supported Au_25_(glutathione)_18_ catalysts for benzyl alcohol oxidation

**DOI:** 10.3762/bjnano.10.21

**Published:** 2019-01-18

**Authors:** Zahraa Shahin, Hyewon Ji, Rodica Chiriac, Nadine Essayem, Franck Rataboul, Aude Demessence

**Affiliations:** 1 Univ Lyon, Université Claude Bernard Lyon 1, CNRS, Institut de Recherches sur la Catalyse et l’Environnement de Lyon (IRCELYON), Villeurbanne, France; 2 Univ Lyon, Université Claude Bernard Lyon 1, CNRS, Laboratoire des Multimatériaux et Interfaces (LMI), Villeurbanne, France

**Keywords:** benzyl alcohol oxidation, glutathione, gold nanoclusters, partial defunctionalization, supported catalyst, zirconium oxide nanoparticles

## Abstract

Au_25_(SG)_18_ (SG – glutathione) clusters deposited on ZrO_2_ nanoparticles have been used as a catalyst for benzyl alcohol oxidation. Calcination was performed at different temperatures to study the ligand and particle size effect on the catalytic activity. In contrast to most gold nanoclusters which have to be completely defunctionalized for maximum catalytic activity, the partially defunctionalized Au_25_(SG)_18_@ZrO_2_ catalyst, thermally treated at 300 °C, exhibits full conversion of benzyl alcohol within 15 h under atmospheric pressure with 94% selectivity towards benzaldehyde.

## Introduction

Since Haruta’s discovery of the catalytic activity of gold nanoparticles (GNPs), GNPs have been of great interest in chemistry, dispersed on metal oxides and in CO oxidation reaction [1]. Today, GNPs of diameter less than 10 nm are known to be a remarkable, heterogeneous catalyst, capable of catalyzing a wide range of reactions including hydrocarbon combustion [2], direct synthesis of hydrogen peroxide by the hydrogenation of O_2_ [3], ozone decomposition [4], selective oxidation reactions [5–8] and so on. However, a debate regarding the particle size effect on the catalytic activity and the concerns related to the synthesis and stabilization of monodisperse GNPs is still ongoing [9–10].

Gold thiolate nanoclusters (GNCs) hold promise due to (i) their atomically well-defined structure with a precise formula, Au*_n_*(SR)*_m_*, in the range of *n* = 10 [11] to 279 [12], i.e., from 1 to 2.2 nm and (ii) for some of them their crystallographically solved structures [13–15]. The Au_25_(SR)_18_ gold thiolate cluster, the captain of the gold nanoclusters ship, is a thermodynamically stable cluster consisting of 25 gold atoms and protected by 18 thiolate ligands [16]. This gold thiolate cluster has been widely studied for its high potential in different domains of chemical sensing, bioimaging, biotherapy and catalysis. As a catalyst, GNCs, and mostly Au_25_(SR)_18_ gold thiolate clusters, have shown high activity for different reactions such as liquid or gas phase oxidation, hydrogenation, C–C coupling and electro/photocatalysis [13].

Based on different studies, it is has been shown that the presence or absence of the thiolate ligand affects the catalytic activity and selectivity of gold thiolate clusters [17–18]. For example, high activity in the aerobic epoxidation of *trans*-stilbene was observed using non-calcined Au_25_(SPhNH_2_)_17_@SBA-15, whereas upon calcination, its activity decreased [19]. In contrast, fully defunctionalized clusters are essential for CO [20], alcohol [17,21], cyclohexane [22] and styrene [23–24] oxidation, as well as nitrobenzene hydrogenation [24]. Recently, a partially calcined Au_38_(2-phenylethanethiolate)_24_ cluster supported on activated carbon (AC) exhibited high efficiency in glucose oxidation [25]. The full defunctionalization at higher temperature usually induces an increase in particle size and decrease of the catalytic activity [26].

Benzyl alcohol oxidation is a model reaction generally used to test the catalytic activity of gold-based materials [27–31]. In the literature, different gold thiolate clusters grafted on different supports were used to selectively oxidize benzyl alcohol. Thus, Au_25_(6-mercaptohexanoic)acid_18_@HAP (HAP – hydroxyapatite) was defunctionalized either by using *tert*-butyl hydroperoxide or by calcination at 300 °C and showed, in both cases, incomplete conversion of the alcohol (46%) under 5 bar of O_2_, at 30 °C and in the presence of a base [32]. Another heterogeneous catalyst, Au_25_(dodecanethiolate)_18_ deposited on porous carbon nanosheets, has been thermally treated at 500 °C for 4 h and showed full conversion of benzyl alcohol into mostly benzoic acid, under 1 atm of O_2_ at 30 °C using a base [17]. In a previous study by our group, Au_25_(SPhNH_2_)_17_@SBA-15, calcined at 400 °C to fully remove the ligands, induced the full conversion of benzyl alcohol after a couple of hours in toluene at 80 °C with a base and under atmospheric conditions [21]. Using O_2_ as an oxidant under atmospheric conditions is a limitless and inexpensive oxidizing agent and allows for a sustainable transformation. Nevertheless, in the last example, the mesoporous silica support exhibits low stability in basic media.

In the context of using atmospheric conditions for the oxidation of benzyl alcohol and a stable support, we present in this work the catalytic activity of a new composite material: Au_25_(SG)_18_ clusters (SG – glutathione) supported on ZrO_2_ nanoparticles. The interest in using ZrO_2_ comes from its high physical and chemical stability, along with its ability to form nanoparticles for high dispersion of the gold nanoclusters [24]. In this work, we synthesized Au_25_(SG)_18_@ZrO_2_ (A), a composite material, and studied the calcination effect to control the defunctionalization of the clusters on the activity and selectivity of the heterogeneously catalyzed benzyl alcohol oxidation.

## Results and Discussion

### Catalyst characterization

A Au_25_(SG)_18_@ZrO_2_ composite material (A), with a theoretical gold loading of 1 wt % Au, was prepared by depositing Au_25_(SG)_18_ gold clusters on ZrO_2_ nanoparticles.

Zirconium hydroxide, Zr(OH)_4_, was used as a precursor for the ZrO_2_ nanoparticles. Zr(OH)_4_ was calcined at 550 °C for 12 h under air at a rate of 2 °C/min. The powder X-ray diffraction (PXRD) pattern of the obtained powder indicated the presence of two crystallographic phases of ZrO_2_, monoclinic and tetragonal (Figure S1, Supporting Information File 1). The transmission electron microscopy (TEM) image shows that the ZrO_2_ particles have a diameter of around 50 nm.

Au_25_(SG)_18_ was synthesized according to a reported method [33]. The characterization of the clusters by UV–vis spectroscopy shows two absorption peaks centered at 450 and 650 nm, which correspond to the electronic transitions typical of this molecular composition (Figure S2, Supporting Information File 1) [34–35]. The PXRD of the clusters exhibited an intense reflection at 5.01°, corresponding to a center-to-center distance between two clusters of 1.76 nm, by applying Bragg’s law (Figure S3, Supporting Information File 1) [36]. This distance is in good agreement with the expected size of Au_25_(SG)_18_ including the ligands. In addition, the broad peak at 37° corresponds to the ultra-small Au_25_ gold core and confirms the absence of large gold nanoparticles or bulk gold.

Impregnation of the clusters on ZrO_2_ nanoparticles was done by adding ZrO_2_ powder to an aqueous solution of Au_25_(SG)_18_, stirred for 15 min and then centrifuged to collect the powder without further washing. The composite material Au_25_(SG)_18_@ZrO_2_ comprised of 0.7% Au was calcined at different temperatures (200, 300 and 400 °C) to gradually remove the ligands. This calcination process induces a change in color from beige for Au_25_(SG)_18_@ZrO_2_ to pink for the calcined samples. Before and after each calcination step, the PXRD patterns of the obtained materials showed no change from the ZrO_2_ diagrams and no indication of reflection of bulk gold (Figure S4, Supporting Information File 1). These observations mean that there is no modification of the support and that the quantity of gold is too small to be detected.

**Figure 1:**
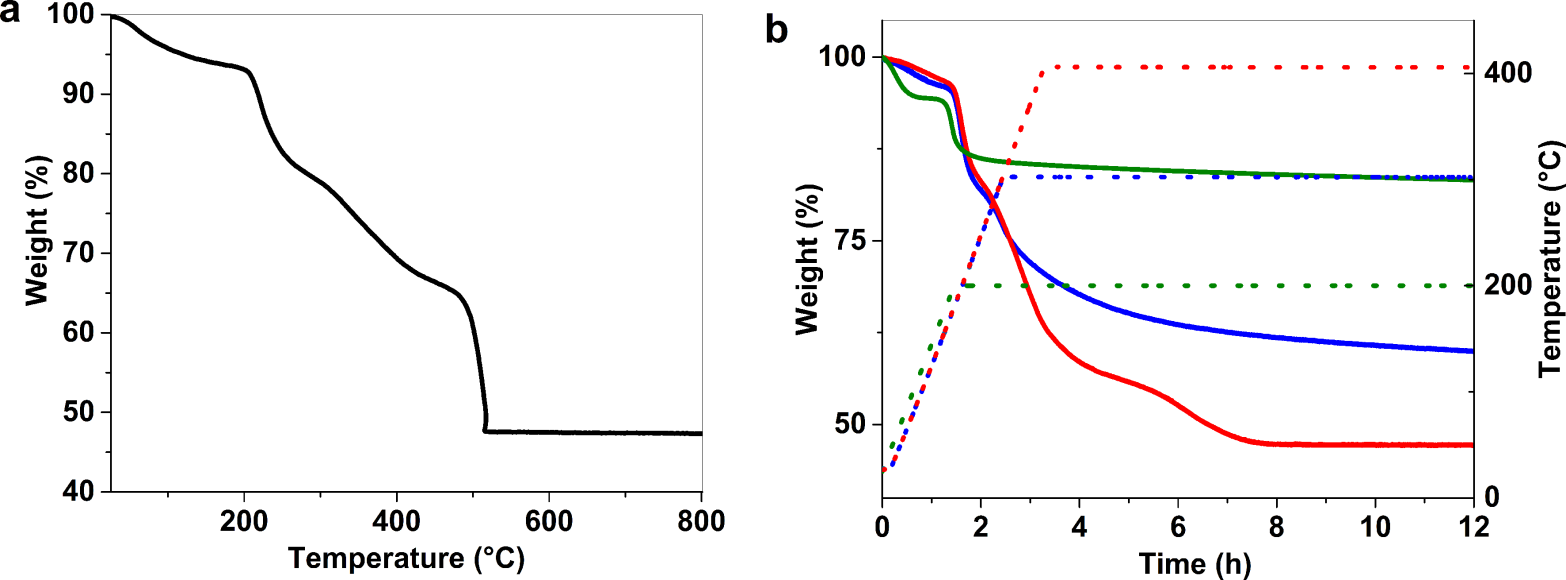
(a) TGA experiment on Au_25_(SG)_18_ under air at 10 °C/min. (b) Isotherm experiments on Au_25_(SG)_18_ under air at 200 °C (green), 300 °C (blue) and 400 °C (red) for 12 h. Solid lines represent the weight (%) and dotted lines represent the heating ramp.

**Figure 2:**
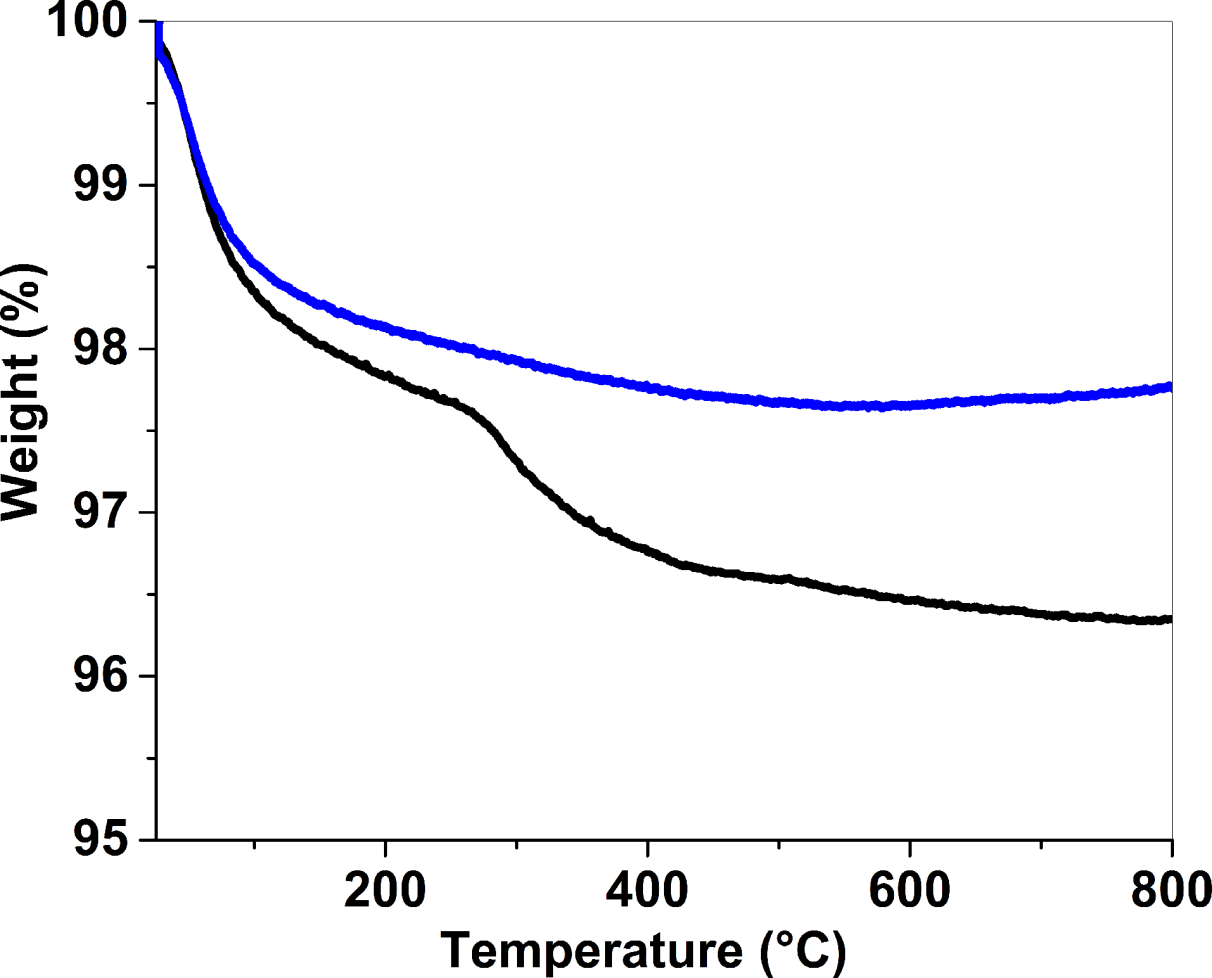
TGA experiments on Au_25_(SG)_18_ deposited on ZrO_2_ (black) and ZrO_2_ alone (blue) carried out under air at 10 °C/min.

Thermal studies of the materials were done by thermogravimetric analysis (TGA) under air on pure Au_25_(SG)_18_ gold clusters, gold clusters deposited on ZrO_2_ and ZrO_2_ alone. From the TGA curve of the clusters, a first gradual weight loss of 6.5% is observed before 200 °C, corresponding to the evaporation of the solvent (Figure 1a). Then a second gradual weight loss of 46.2% happens up to 500 °C and the remaining gold is 47.3%. The actual percentage of glutathione (49.4%) is a little less than the calculated value (53.0%). This difference in thiolate ligand may be due to the early decomposition of the molecules before 200 °C or to the presence of impurities such as bigger clusters [37]. Isotherm analysis was performed at 200 °C, 300 °C, and 400 °C, with the temperatures kept constant for 12 hours under air, in order to simulate the calcination procedures. For the isotherm analysis at 200 °C, 300 °C, and 400 °C, the final loss reached 17.0%, 40.8% and 52.9%, respectively, corresponding to a partial calcination of 36.5% at 300 °C and to a complete removal of the ligand (77.1%) at 400 °C (Figure 1b). It is interesting to note that the complete calcination of the ligands at 400 °C is reached after almost 8 h of heating, suggesting that a heating ramp of at least 8 hours is required to completely remove the glutathione molecules from the gold surface. For the TGA of the ZrO_2_ support, 2.4% weight loss was observed at low temperature that corresponds to trace water (Figure 2). After the deposition of 1 wt % Au using Au_25_(SG)_18_ on ZrO_2_, a first weight loss of 2.4% is observed and a second weight loss of 1.3% from 250 °C is also seen and fits well with the decomposition of the glutathione molecules (Figure 2).

**Figure 3:**
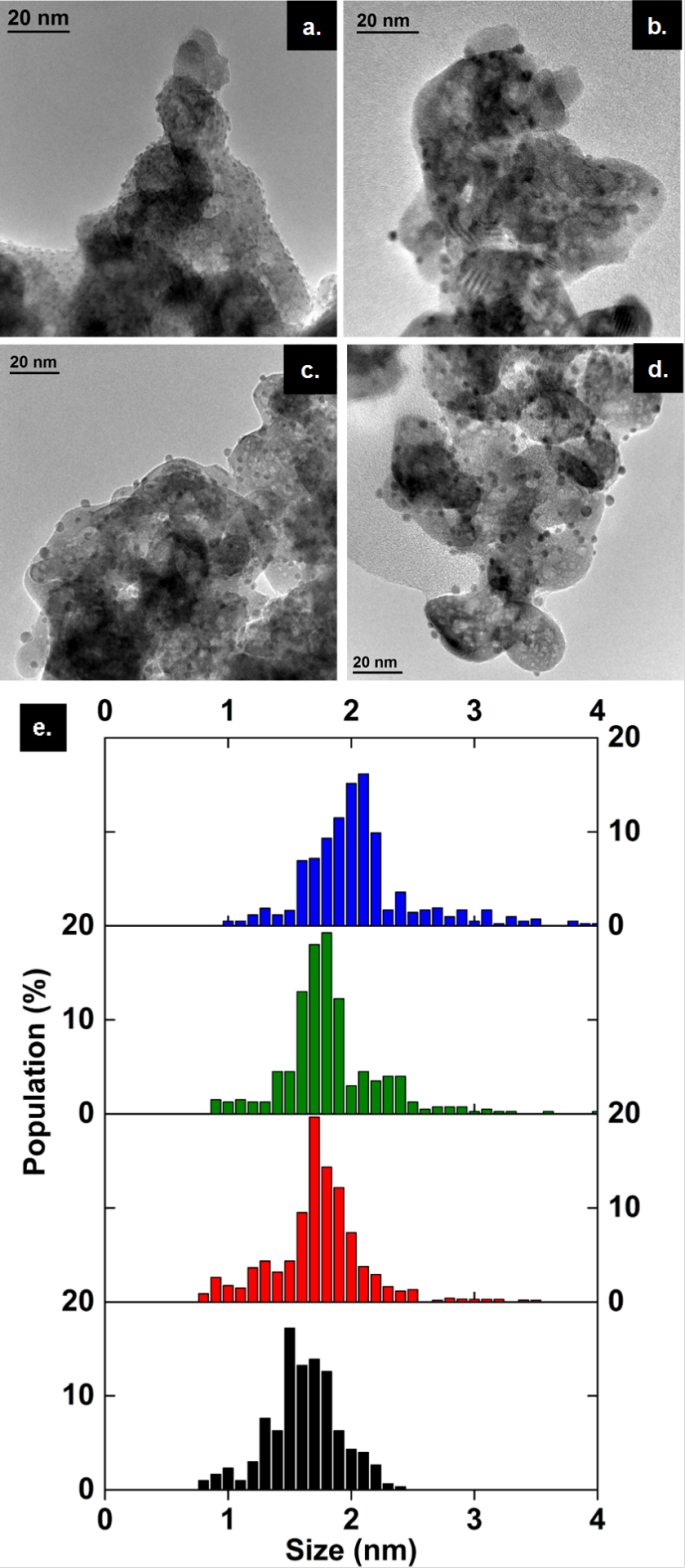
TEM images of Au_25_(SG)_18_@ZrO_2_ (Au 0.7 wt %) (a) before calcination, sample (A), (b) calcined at 200 °C for 4 hours under air, sample (A_200_), (c) calcined at 300 °C for 4 hours under air, sample (A_300_), and (d) calcined at 400 °C for 12 hours under air, sample (A_400_), and (e) the size distribution of the composites – (A) in black, (A_200_) in red, (A_300_) in green and (A_400_) in blue.

The influence of calcination temperature on the particle size of the clusters deposited on ZrO_2_ was evaluated from the TEM images and size distribution analysis (Figure 3). The composite material Au_25_(SG)_18_@ZrO_2_ is named (A) and (A_200_), (A_300_) and (A_400_) after calcination under air at 200 and 300 °C for 4 h and 400 °C for 12 h, respectively. Sample (A) exhibits homogeneous clusters of size 1.6 ± 0.3 nm (Figure 3a,e), being close to the expected Au_25_ diameter estimated from the crystal structure (1 nm) [38]. For (A_200_) the mean particle size is 1.6 ± 0.7 nm and approximately the same for (A_300_) at 1.7 ± 0.5 nm (Figure 3b,c,e). For (A_400_), the particle size increased to 2.0 ± 0.7 nm, which may be due to the sintering of the bare Au_25_ gold cores (Figure 3d,e). In general, supported gold thiolate clusters are known to grow when calcined at high temperature [24], except when they are inserted in a porous material, such as SBA-15 [21], or loaded with a very small quantity of clusters [22]. Here we note that the gold clusters maintain a diameter of around 2 nm or below, with a narrow size distribution, upon calcination at temperatures up to 400 °C with 0.7 wt % Au loading.

### Catalytic performance

**Scheme 1:**
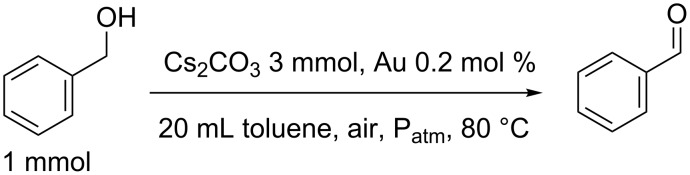
Benzyl alcohol oxidative dehydrogenation under standard conditions.

The catalytic activity of Au_25_(SG)_18_@ZrO_2_, calcined at different temperatures, was studied for the oxidative dehydrogenation of benzyl alcohol to benzaldehyde in the presence of an excess of base (Cs_2_CO_3_, 3 eq.) at 80 °C and under atmospheric conditions (Scheme 1). Before observing the influence of calcination of thiolates on the activity of the gold catalysts, a blank and the support alone were run to confirm the catalytic activity of the gold catalyst. Since there was no benzyl alcohol conversion and no formation of benzaldehyde in both cases, it was deduced that the reaction conditions, such as temperature or atmospheric O_2_ did not have any catalytic role in the oxidation reaction.

### Influence of the calcination temperature

**Figure 4:**
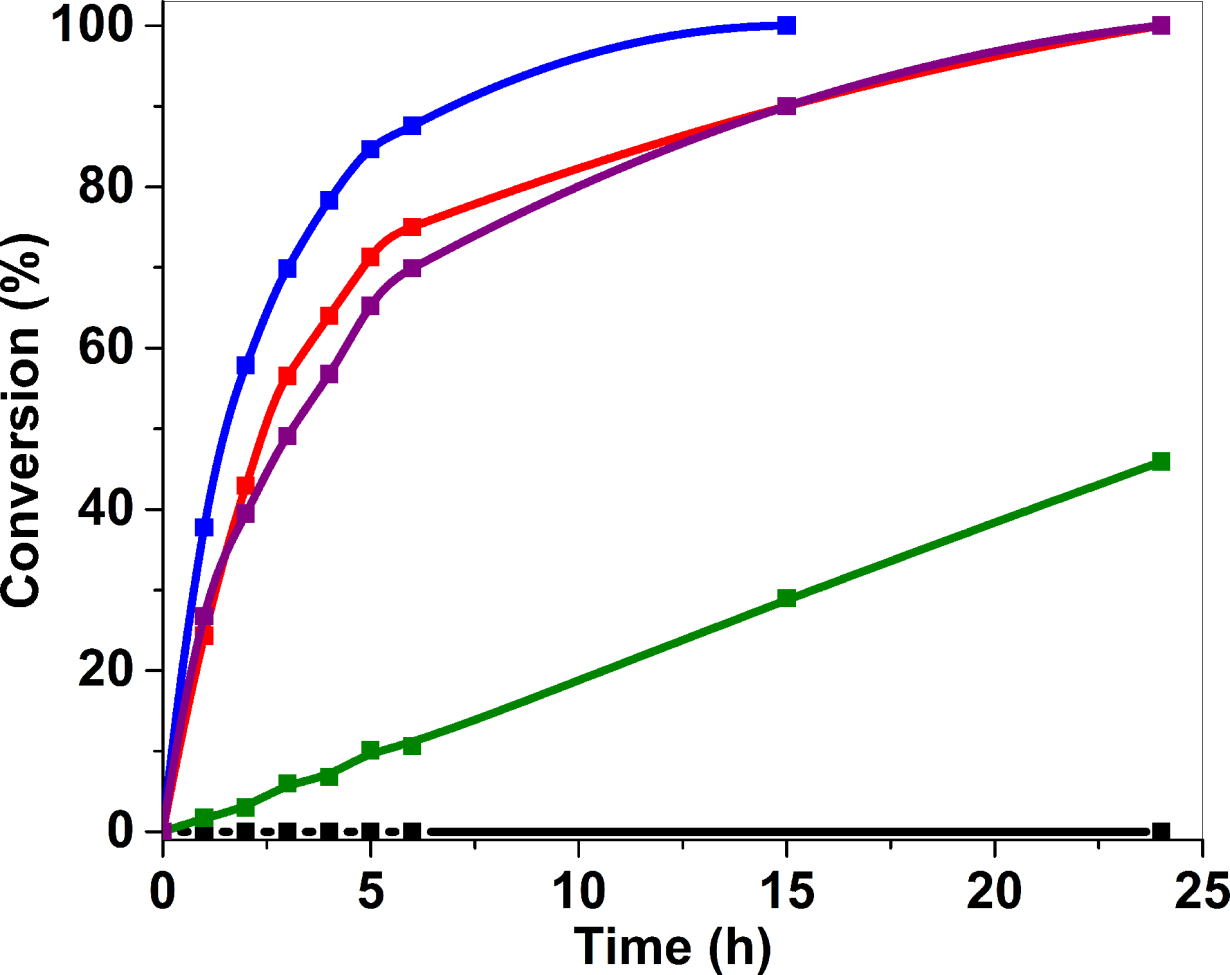
Monitoring over time of benzyl alcohol oxidative dehydrogenation conversion with Au_25_(SG)_18_@ZrO_2_ before calcination (black), after calcination at 200 °C for 4 hours under air, (A_200_) (green), at 300 °C for 4 hours under air, (A_300_) (blue), at 400 °C for 12 hours under air, (A_400_) (red) and compared to AuNP@ZrO_2_ (purple).

Au_25_(SG)_18_@ZrO_2_ (A) was inactive and unable to oxidize benzyl alcohol to benzaldehyde. Despite the well-dispersed, homogenously small-sized gold particles, as seen from the TEM image (Figure 3a) and the size distribution graph (Figure 3e), their catalytic activity was likely to be affected by the presence of the thiolate ligands. The same behavior was observed for the untreated Au_25_(SPhNH_2_)_17_@SBA-15, which did not show any activity for benzyl alcohol oxidation [21]. For (A_200_), 64.2% of the thiolate ligands remained, and benzyl alcohol conversion reached 50% after 12 h with an initial turn over frequency (TOF) of 10 h^−1^, which was very low compared to that of (A_300_). The latter had 46.5% of the thiolate ligands remaining and only 1.5 h were needed to reach 50% conversion with a TOF = 261 h^−1^, showing that the partial calcination had improved the catalyst activity. For (A_400_), for which no thiolate ligands remained, 2.4 h were needed to reach 50% conversion with a TOF = 123 h^−1^ (Figure 4 and Table 1).

**Table 1:** Catalytic performance of Au_25_(SG)_18_@ZrO_2_ based catalysts (2 µmol Au) in the oxidative dehydrogenation of benzyl alcohol in toluene at 80 °C (1 atm of air): 25%, 50% and 90% conversion times (*t*), benzaldehyde selectivity at half conversion (Sel 50%), turn over frequency (TOF) and gold particle size measured by TEM before the catalytic test. ND: Not determined.

Sample	Catalyst	*t*_25%_ (h)	*t*_50%_ (h)	*t*_90%_ (h)	Sel_50%_ (%)	TOF (h^−1^)	Average AuNP diameter (nm)

(A)	Au_25_(SG)_18_@ZrO_2_	ND	ND	ND	ND	–	1.6 ± 0.3
(A_200_)	(A) calcined at 200 °C	6	12	21.6	80	10	1.6 ± 0.7
(A_300_)	(A) calcined at 300 °C	0.6	1.5	6.8	94	261	1.7 ± 0.5
(A_400_)	(A) calcined at 400 °C	1	2.4	15	100	123	2.0 ± 0.7
(B)	AuNP@ZrO_2_	1	3	15	100	144	2.7 ± 1.5

The increase in catalytic activity from (A) to (A_200_) and the highest TOF (261 h^−1^) in the case of (A_300_), is explained by the increase of defunctionalization of the supported thiolate clusters, which triggered the catalytic activity. However, the decrease in catalytic activity of (A_400_), with a lower TOF value (123 h^−1^), though it was fully defunctionalized, is related to the sintering of the gold nanoparticles, where bigger 2.0 ± 0.7 nm particles were observed on the TEM images. This means that both the defunctionalization and the particle size affect the catalytic activity of the composite material. A balance between both is required to have maximum activity, as in (A_300_), where 46.5% of the thiolate ligands remained, triggering gold activity and keeping small sized particles at 1.7± 0.5 nm. Therefore, partially calcined clusters did not inhibit high catalytic activity, in contrast, it was enhanced, which was similar to a recent reported work [25].

**Figure 5:**
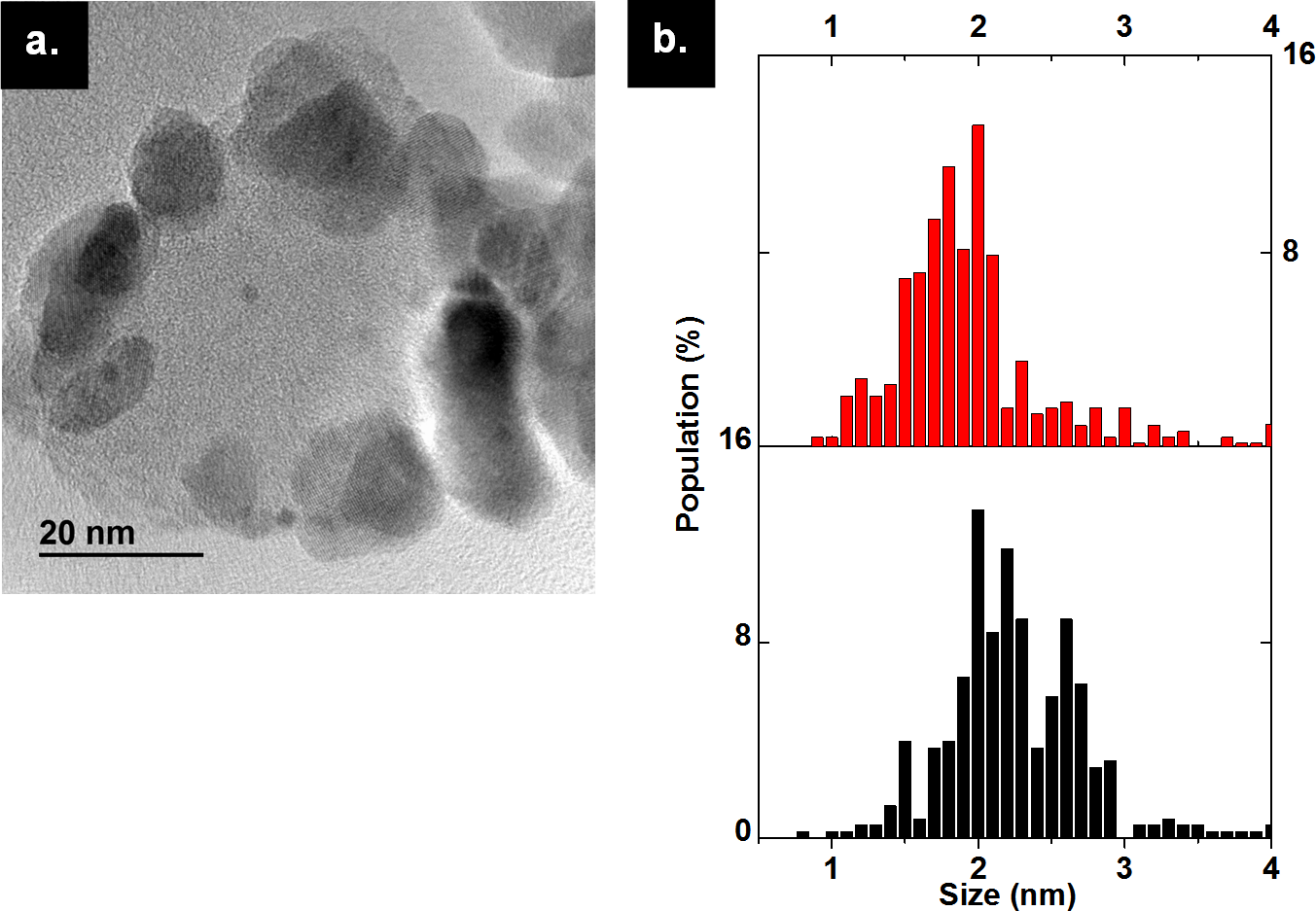
(a) TEM image of AuNP@ZrO_2_ prepared by the deposition-precipitation method (B). (b) Comparison of size distribution of Au_25_(SG)_18_@ZrO_2_ calcined at 400 °C for 12 hours under air (A_400_) in red and AuNps@ZrO_2_ (B) in black.

The catalyst performance was compared to a catalyst synthesized by the deposition-precipitation method of gold nanoparticles on ZrO_2_ nanoparticles, compound (B). The average particle size of (B), measured by TEM images, is 2.7 ± 1.5 nm, higher than that of the gold particles obtained in (A_400_) after full calcination (Figure 5). Compound (B) showed 50% conversion of benzyl alcohol in 3 h, a value close to that obtained with (A_400_), having slightly higher initial TOF (144 h^−1^). They both reached 90% conversion after 15 h. This shows that when gold nanoparticles have a diameter more than 2 nm, they act in a similar catalytic manner, but still have slower catalytic activity compared to the partially calcined composite material (A_300_) (Table 1).

At the selectivity level of 50% (Sel_50%_) conversion toward benzaldehyde, an increase with the increase of calcination temperature was observed for compound (A). The Sel_50%_ for (A_200_) was 80%, less than that of (A_300_), at 94%, which was also lower than the Sel_50%_ of (A_400_) and (B) at 100% (Table 1). This means that having pure gold without any organic linker is necessary to have high selectivity toward benzaldehyde, but still the partially calcined composite material (A_300_), with comparable selectivity of 94%, to (A_400_) and (B), resulted in the best activity with highest TOF = 261 h^−1^.

Compared to previous studies, Au_25_(SC_12_H_25_)_18_ supported on hierarchically porous carbon nanosheets [17] and Au_25_(SPhNH_2_)_17_ supported on SBA-15 [21], both calcined at 400 °C, showed 67% and 68% of selectivity for benzaldehyde, respectively. Thus, the 100% selectivity for benzaldehyde of Au_25_(SG)_18_ over ZrO_2_ when calcined at 400 °C may result from the different compositions of the clusters or the effect of the type of support that can be involved in the oxidation mechanism or their different morphologies, as porous materials for the carbon nanosheets and the silica, and nanoparticles for ZrO_2_.

### Effect of the reaction temperature

**Figure 6:**
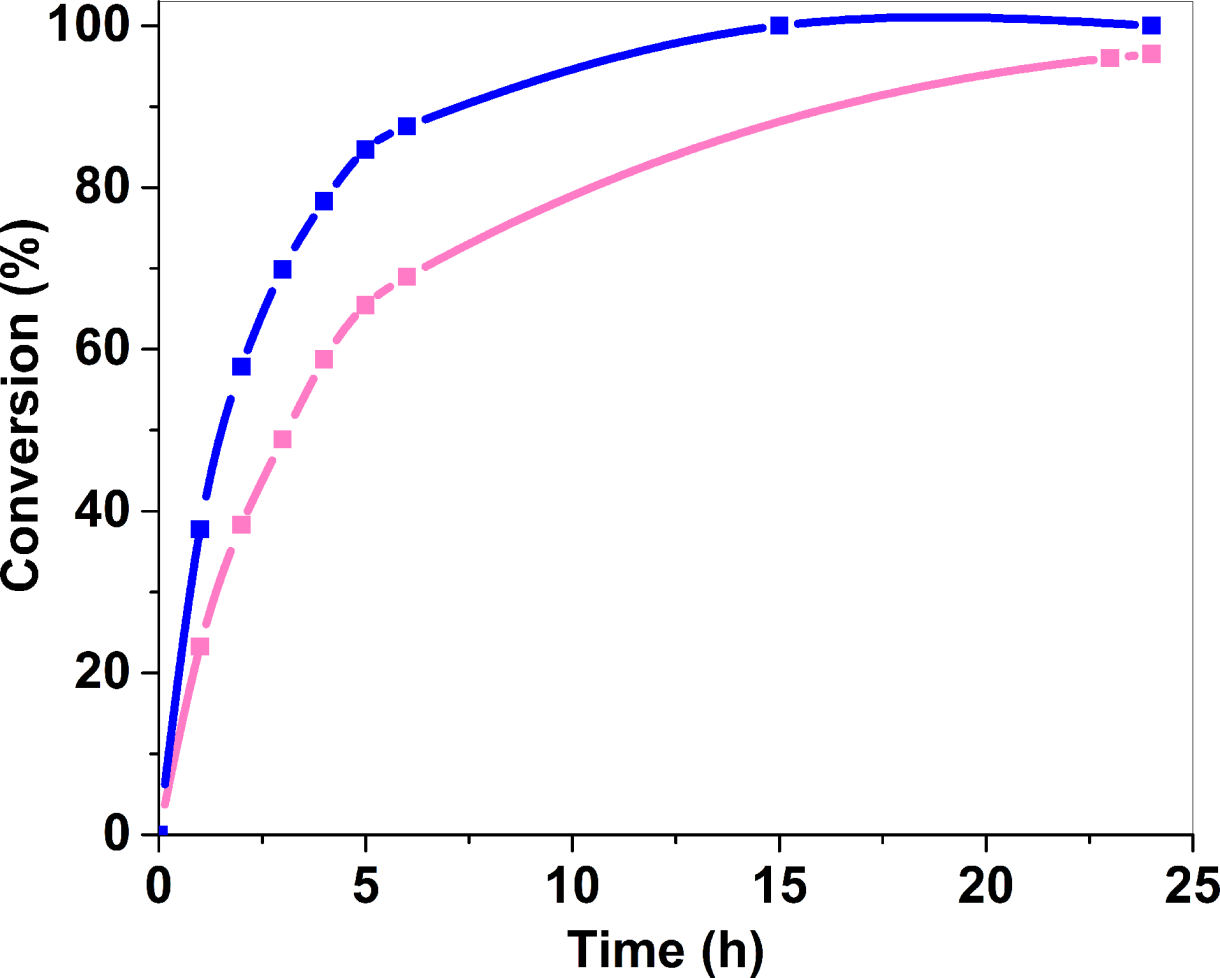
Monitoring over time of benzyl alcohol oxidative reaction with Au_25_(SG)_18_@ZrO_2_ calcined at 300 °C for 4 hours under air (A_300_) at 60 °C (pink), and at 80 °C (blue).

In general, the oxidation of benzyl alcohol is performed under harsh conditions of temperature and pressure without a catalyst [39]. Gold-based catalysts perform this oxidation under milder conditions [31]. The reaction using (A_300_) as a catalyst, was performed at two different temperatures, 60 °C and 80 °C, with all other experimental conditions being the same. Such relatively low temperatures showed no thermal conversion of benzyl alcohol without catalyst. The conversion curves clearly showed that the increase of the temperature of 20 °C favors the benzyl alcohol conversion (Figure 6). At 60 °C, the time necessary to reach 50% conversion is 3 h, whereas it is 1.5 h at 80 °C. Besides, the Sel_50%_ increased from 75% to 94% with temperature, suggesting that the faster the reaction rate, the higher the benzaldehyde selectivity (Table 2).

**Table 2:** Catalytic performance of (A_300_) catalyst (2 µmol Au) in the oxidative reaction of benzyl alcohol in toluene at 80 °C and 60 °C (1 atm of air): 25%, 50% and 90% conversion time (*t*), benzaldehyde selectivity at half conversion (Sel 50%) and turn over frequency (TOF).

	Reaction temperature (°C)	*t*_25%_ (h)	*t*_50%_ (h)	*t*_90%_ (h)	Sel_50%_ (%)	TOF (h^−1^)

(A_300_)	80	0.6	1.5	6.8	94	261
(A_300_)	60	1.2	3	16	75	101

### Recyclability of the catalyst

**Figure 7:**
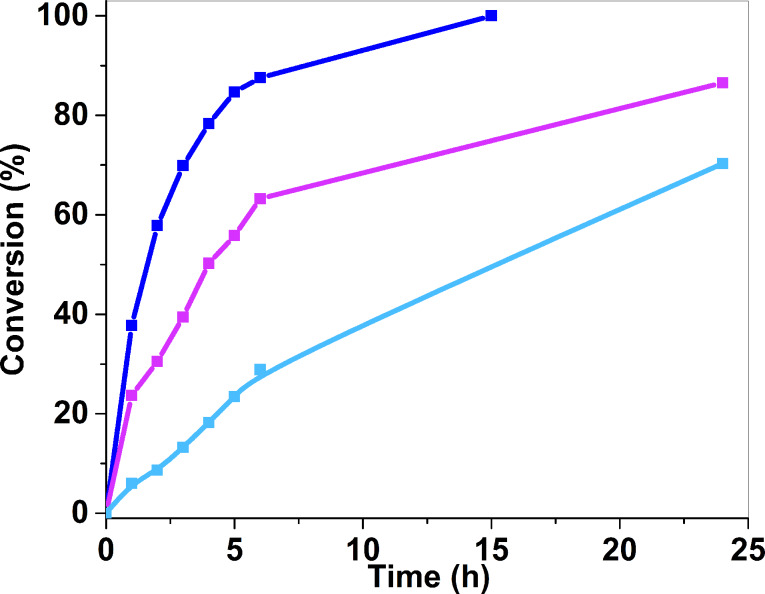
Monitoring over time of benzyl alcohol oxidative dehydrogenation conversion for successive additions of 1 mmol BnOH in the reaction medium (each reaction was carried out during 24 h) using (A_300_) as a catalyst. (A_300_)^1^ represents the conversion (%) while using the catalyst for the first cycle (blue), (A_300_)^2^ for the second cycle (pink) and (A_300_)^3^ for the third cycle (cyan).

**Figure 8:**
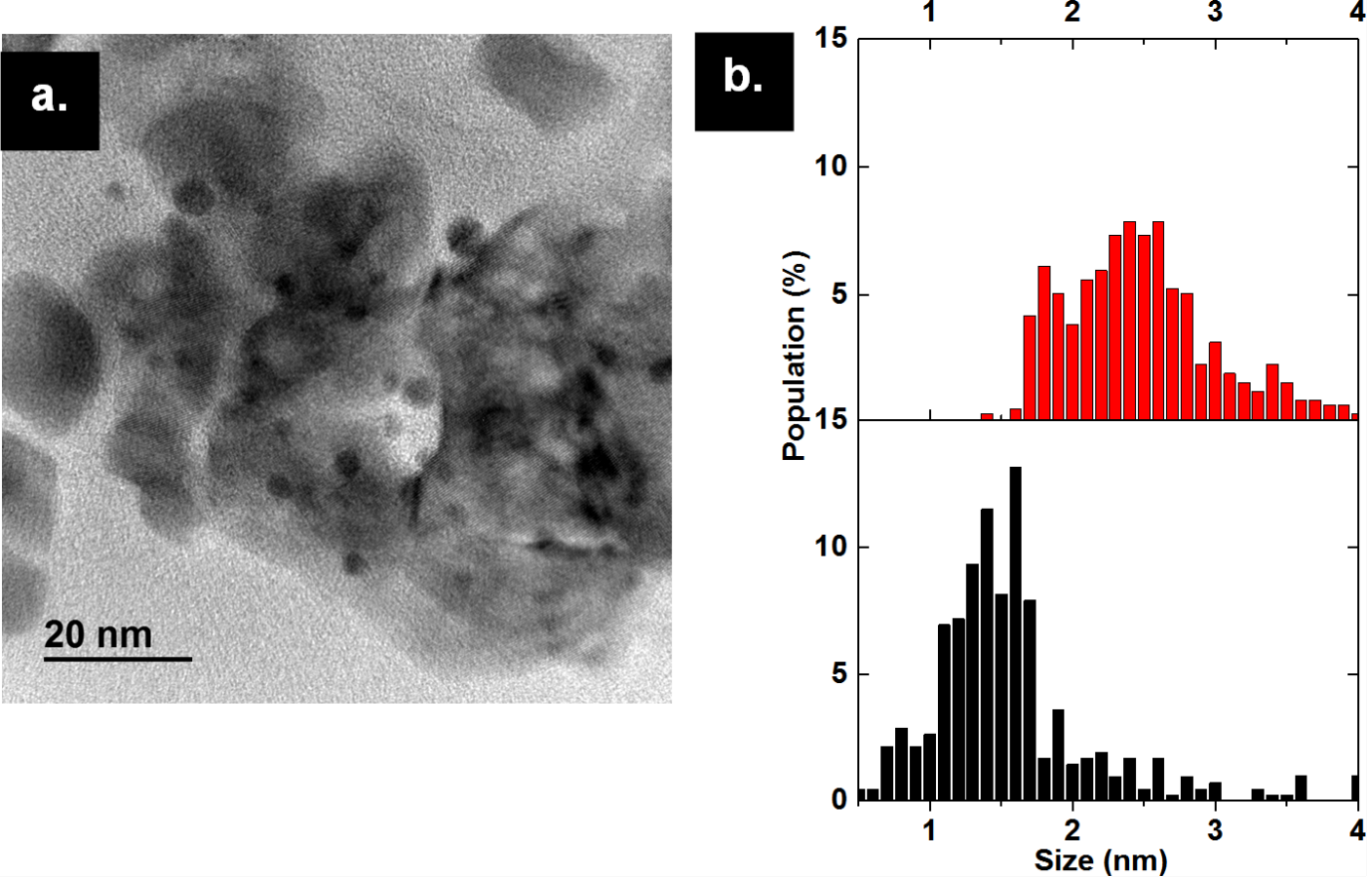
(a) TEM image of (A_300_)^3^ after the third catalytic cycle. (b) Comparison of the size distribution of (A_300_)^3^ in red and (A_300_) before the catalytic test in black.

The recyclability of (A_300_), the catalyst that showed the highest TOF value in the oxidative dehydrogenation of benzyl alcohol, was tested by adding a new portion of benzyl alcohol to the reaction mixture after each cycle. It was observed that after each run, the catalytic stability decreased, giving full conversion in the first cycle (A_300_)^1^, 86.6% conversion in the second cycle (A_300_)^2^ and 70.3% in the third cycle (A_300_)^3^, after 24 h of reaction (Figure 7). This decrease in the catalytic activity is explained by particle aggregation and sintering with time. The particle size after the third cycle in (A_300_)^3^ was 2.8 ± 0.8 nm (Figure 8).

## Conclusion

Successfully supported Au_25_(SG)_18_ clusters on ZrO_2_ nanoparticles was used as a catalyst, after activation, in the oxidative dehydrogenation of benzyl alcohol to benzaldehyde. The effect of the calcination temperature was studied by subsequent calcination steps under different conditions. For partial defunctionalization, activation at 200 °C and 300 °C for 4 h was done under air, whereas the treatment at 400 °C for 12 hours resulted in the complete removal of the thiolate ligands. The influence of the presence of thiolate ligands and the size of the particles was clearly observed during benzyl alcohol conversion, where the full conversion was observed after 15 h with the catalyst partially defunctionalized at 300 °C under air for 4 hours with particle of 1.7 ± 0.5 nm diameter. This study confirmed that the activity and selectivity of supported Au_25_(SG)_18_ clusters are highly efficient for oxidation reactions carried out under mild conditions of ambient atmosphere and temperature (80 °C), and most importantly do not require the complete removal of the thiolate ligands.

## Experimental

### Chemicals

Tetrachloroauric acid trihydrate (HAuCl_4_^.^3H_2_O, ≥99.9% trace metal basis), sodium borohydride (NaBH_4_, ≥98.0%), benzyl alcohol and dodecane (≥99%) were purchased from Sigma-Aldrich. L-glutathione (HSG, +98%) and cesium carbonate (99%, metal basis) were obtained from Alfa Aesar, Methanol (HPLC grade) from VWR International, and toluene from Emsure. Zirconium oxide (ZrO_2_) was prepared from Zr(OH)_4_ calcined at 550 °C for 12 hours under air flow at a rate of 2 °C/min. All chemicals were used without further purification. All glassware were washed with aqua regia and rinsed with ethanol. Ultrapure water (18 MΩ) was used in all experiments.

### Characterization techniques

Powder X-ray diffraction (PXRD) was carried out on a Bruker D8 Advance A25 diffractometer using Cu Kα radiation. Small-angle X-ray scattering was recorded between 0.45° and 7° (2θ) with 0.01° steps and 2 s per step. Standard acquisition was recorded between 4° and 80° (2θ) with 0.02° steps and 0.5 s per step.

Thermogravimetric analysis (TGA) was performed with a TGA STARE system from Mettler Toledo Thermobalance MX1. Around 2 mg of sample was heated from 25 °C to 800 °C at a rate of 10 °C/min in a 70 µL alumina crucible, under air.

For isothermal TGA, the samples were heated at a rate of 2 °C/min from 25 °C to the final targeted temperature (200 °C, 300 °C, and 400 °C) in a 70 µL alumina crucible, under air. The final temperature was maintained for 12 hours.

Transmission electron microscopy (TEM) was carried out on a JEOL 2010 LaB_6_ microscope operating at 200 kV. The samples were prepared on a copper grid for analysis. The measurement of the diameter of the particles was done by using the TEM images, where the diameter of each particle was measured by hand by using Image J software. A minimum number of particles of 300 was measured to get a distribution.

Gas chromatography was carried out on a Shimadzu GC-2010 device using a 30 m × 0.25 mm × 0.25 µm column programmed from 30 °C to 180 °C, injector and FID detector set at 220 °C, and using N_2_ as carrier gas. External calibration was carried out by injecting distinct standard solutions of benzyl alcohol and benzaldehyde with dodecane.

UV–visible spectroscopy was performed with Agilent UV 8453 UV–visible spectrometer, with a deuterium discharge lamp as the radiation source for ultraviolet wavelength region and a tungsten lamp for the visible and short wave near-infrared wavelength region. Water was used as the blank.

### Synthesis of Au_25_(SG)_18_

Au_25_(SG)_18_ clusters were synthesized following a previously reported synthesis procedure with some modifications [33]. In a 100 mL round-bottom flask, 0.25 mmol HAuCl_4_^.^3H_2_O was dissolved in 50 mL methanol under stirring at 1500 rpm in an ice bath. Then, 1 mmol glutathione was rapidly added to the flask, and the mixed solution was left stirring for 30 minutes. The color of the mixed solution gradually changed from clear yellow to transparent. Meanwhile, the NaBH_4_ solution was prepared by dissolving 2.5 mmol NaBH_4_ in 12.5 mL ice-cold water, which was rapidly added to the mixed solution. An obvious color change to dark brown was observed after the addition of NaBH_4_. The reaction was allowed to proceed under stirring at 1500 rpm in an ice bath for 1 hour, and UV–vis spectra were collected at 45 minutes into the reaction. The product was purified by repeated centrifugation (10000 rpm, 15 minutes) and was washed several times with methanol (5000 rpm, 15 minutes). The obtained product was dried under vacuum at room temperature, and was kept in the refrigerator until the second part. In the second part of the synthesis, the product was dissolved in 12.5 mL water and 0.5 mmol glutathione was added. The mixture was left stirring at 60 rpm and heated with an oil bath at 55 °C for 4 hours. The final product Au_25_(SG)_18_ was filtered, isolated by precipitation with methanol and centrifuged (10000 rpm for 15 minutes), washed several times with methanol (5000 rpm, 15 minutes), and was air-dried.

### Synthesis of the composite material Au_25_(SG)_18_@ZrO_2_

#### Gold cluster deposition

Au_25_(SG)_18_ cluster deposition was performed using a wet impregnation method. Gold clusters, with a mass of 10 mg corresponding to a theoretical loading of 1 wt % Au, and 500 mg of support (ZrO_2_) were dispersed in 5 mL of water, swirled, and left for 15 minutes. The prepared catalyst (A) was recovered by centrifugation (4000 rpm, 10 minutes) after the addition of small amounts of ethanol, and was followed by drying under air.

#### Calcination

Calcination was performed on Au_25_(SG)_18_@ZrO_2_. Around 100 mg of compounds were heated at 200 °C for 4 hours under air, 300 °C for 4 hours under air, and 400 °C for 12 hours under air, with a rate of 2 °C/min.

#### Synthesis of AuNP@ZrO_2_ by deposition-precipitation

The synthesis of AuNP@ZrO_2_ was done according to a reported protocol [40]. An aqueous solution of tetrachloroauric acid trihydrate (1.5% by mass, in 10 mL H_2_O) was added dropwise to ZrO_2_ (1 g) dispersed in 30 mL H_2_O while stirring at 400 rpm at room temperature. A yellow solution was obtained. NaOH (0.5 M) was used to adjust the pH at 9, where the solution then turned transparent. The mixture was kept stirring at 400 rpm for 1 h at room temperature. The temperature was then increased up to 80 °C and left stirring for 2 h while keeping pH 9. The reaction was set back at room temperature and left overnight. The product was filtered, dried at 110 °C for 30 minutes, calcined at 350 °C for 4 h under air, then reduced under H_2_ flow at 350 °C for 2 h. The final powder had dark pink-purple color and named (B).

#### Benzyl alcohol oxidation

Catalytic evaluation was carried out following a previously reported procedure [21]. In a two-neck 100 mL round-bottom flask equipped with a condenser and a magnetic stirrer, benzyl alcohol (BnOH, substrate, 1 mmol), cesium carbonate (Cs_2_CO_3_, base, 3 mmol), toluene (solvent, 20 mL) and gold-based catalyst (2 µmol Au) were stirred at 400 rpm at 80 °C under atmospheric air pressure, while connecting the flask to a reflux.

The reactions were monitored by regular samplings (0.2 mL) that were diluted 2 times in the standard dodecane solution (1 wt % in toluene) and were analyzed immediately by gas chromatography. Benzyl alcohol (BnOH) conversion was calculated from the ratio of the number of moles of BnOH converted over the initial quantity of BnOH introduced at the beginning of the reaction. The benzaldehyde (BnAld) yield was calculated from the ratio of the number of moles of BnAld produced over the initial quantity of BnOH introduced at the beginning of the reaction. The selectivity was defined as the ratio of the BnAld yield over BnOH conversion. The given TOF (h^−1^) are the initial TOF, calculated from the ratio of the converted moles of benzyl alcohol over the total moles of the gold content in the catalyst per unit of time.


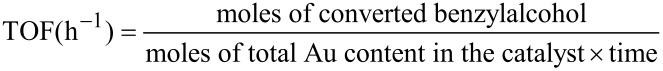


## Supporting Information

File 1 Additional experimental results.

## References

[1] Haruta M, Kobayashi T, Sano H, Yamada N (1987). Chem Lett.

[2] Miao S, Deng Y (2001). Appl Catal, B.

[3] Landon P, Collier P J, Papworth A J, Kiely C J, Hutchings G J (2002). Chem Commun.

[4] Hao Z, Cheng D, Guo Y, Liang Y (2001). Appl Catal, B.

[5] Prati L, Porta F (2005). Appl Catal, A.

[6] Biradar A V, Asefa T (2012). Appl Catal, A.

[7] Hughes M D, Xu Y-J, Jenkins P, McMorn P, Landon P, Enache D I, Carley A F, Attard G A, Hutchings G J, King F (2005). Nature.

[8] Lignier P, Morfin F, Mangematin S, Massin L, Rousset J-L, Caps V (2007). Chem Commun.

[9] Roldan Cuenya B, Behafarid F (2015). Surf Sci Rep.

[10] Zanella R, Giorgio S, Henry C R, Louis C (2002). J Phys Chem B.

[11] Lavenn C, Albrieux F, Tuel A, Demessence A (2014). J Colloid Interface Sci.

[12] Sakthivel N A, Theivendran S, Ganeshraj V, Oliver A G, Dass A (2017). J Am Chem Soc.

[13] Jin R, Zeng C, Zhou M, Chen Y (2016). Chem Rev.

[14] Kurashige W, Niihori Y, Sharma S, Negishi Y (2016). Coord Chem Rev.

[15] Sakthivel N A, Dass A (2018). Acc Chem Res.

[16] Kang X, Chong H, Zhu M (2018). Nanoscale.

[17] Yoskamtorn T, Yamazoe S, Takahata R, Nishigaki J-i, Thivasasith A, Limtrakul J, Tsukuda T (2014). ACS Catal.

[18] Nasaruddin R R, Chen T, Yan N, Xie J (2018). Coord Chem Rev.

[19] Lavenn C, Demessence A, Tuel A (2014). Catal Today.

[20] Wu Z, Jiang D-e, Mann A K P, Mullins D R, Qiao Z-A, Allard L F, Zeng C, Jin R, Overbury S H (2014). J Am Chem Soc.

[21] Lavenn C, Demessence A, Tuel A (2015). J Catal.

[22] Liu Y, Tsunoyama H, Akita T, Xie S, Tsukuda T (2011). ACS Catal.

[23] Huang P, Chen G, Jiang Z, Jin R, Zhu Y, Sun Y (2013). Nanoscale.

[24] Fang J, Li J, Zhang B, Yuan X, Asakura H, Tanaka T, Teramura K, Xie J, Yan N (2015). Nanoscale.

[25] Liu C, Zhang J, Huang J, Zhang C, Hong F, Zhou Y, Li G, Haruta M (2017). ChemSusChem.

[26] Moulijn J A, van Diepen A E, Kapteijn F (2001). Appl Catal, A.

[27] Wang Z, Xu C, Wang H (2014). Catal Lett.

[28] Pina C D, Falletta E, Rossi M (2012). Chem Soc Rev.

[29] Della Pina C, Falletta E (2011). Catal Sci Technol.

[30] Davis S E, Ide M S, Davis R J (2013). Green Chem.

[31] Sharma A S, Kaur H, Shah D (2016). RSC Adv.

[32] Zhang B, Fang J, Li J, Lau J J, Mattia D, Zhong Z, Xie J, Yan N (2016). Chem – Asian J.

[33] Liu X, Wu Y, Li S, Zhao Y, Yuan C, Jia M, Luo Z, Fu H, Yao J (2015). RSC Adv.

[34] Zhu M, Qian H, Jin R (2009). J Am Chem Soc.

[35] Qian H, Zhu M, Andersen U N, Jin R (2009). J Phys Chem A.

[36] Lavenn C, Albrieux F, Bergeret G, Chiriac R, Delichère P, Tuel A, Demessence A (2012). Nanoscale.

[37] Shibu E S, Muhammed M A H, Tsukuda T, Pradeep T (2008). J Phys Chem C.

[38] Heaven M W, Dass A, White P S, Holt K M, Murray R W (2008). J Am Chem Soc.

[39] Sheldon R A, Arends I W C E, ten Brink G-J, Dijksman A (2002). Acc Chem Res.

[40] Schade O R, Kalz K F, Neukum D, Kleist W, Grunwaldt J-D (2018). Green Chem.

