# Supplementary material for: Thermal control of the defunctionalization of supported Au25(glutathione)18 catalysts for benzyl alcohol oxidation
Source: Beilstein J Nanotechnol. 2019 Jan 18;10:228–37. doi: 10.3762/bjnano.10.21 (PMC6350859; doi:10.3762/bjnano.10.21)
Supplement: File 1 — Additional experimental results. [file Beilstein_J_Nanotechnol-10-228-s001.pdf]

BEILSTEIN JOURNAL OF NANOTECHNOLOGY

Supporting Information

for

Thermal control of the defunctionalization of supported Au_{25}(glutathione)$_{18}$ catalysts for benzyl alcohol oxidation

Zahraa Shahin, Hyewon Ji, Rodica Chiriac, Nadine Essayem, Franck Rataboul and Aude Demessence

Beilstein J. Nanotechnol. **2019,** *10,* 228–237. doi:10.3762/bjnano.10.21

Additional experimental results

License and Terms: This is a supporting information file under the terms of the Creative Commons Attribution License (http://creativecommons.org/licenses/by/4.0). Please note that the reuse, redistribution and reproduction in particular requires that the authors and source are credited. The license is subject to the *Beilstein Journal of Nanotechnology* terms and conditions: (https://www.beilstein-journals.org/bjnano)

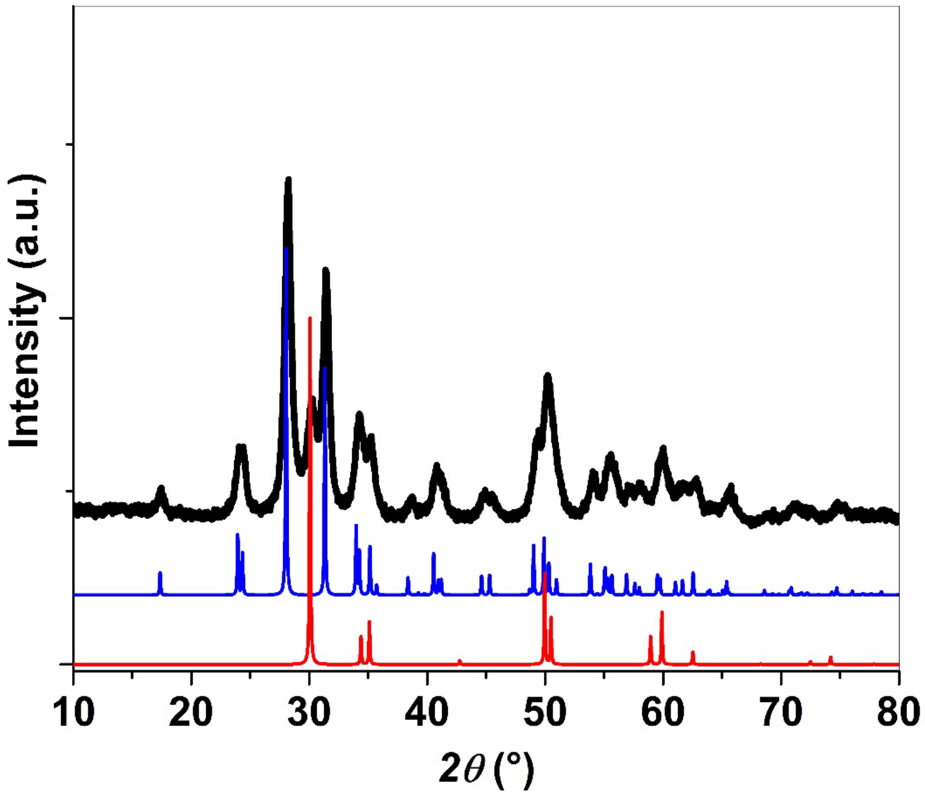

Figure S1: Powder X-ray diffraction patterns of synthesized ZrO_2 (black) and simulated monoclinic (blue) and tetragonal (red) phases of ZrO_2.

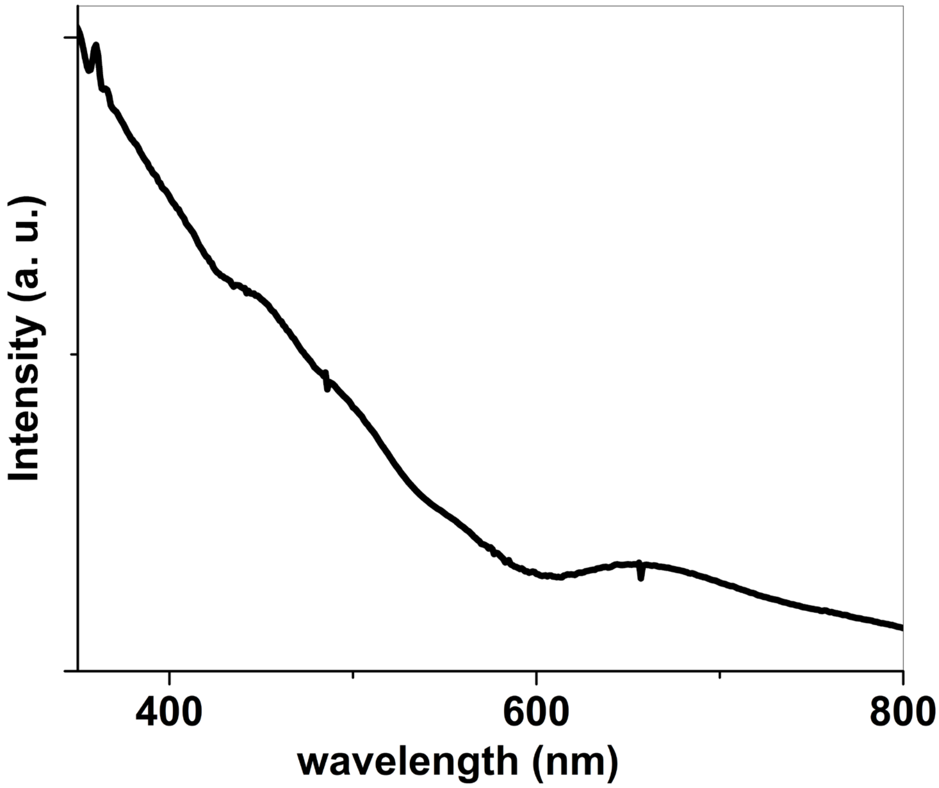

Figure S2: UV-vis spectrum of $Au_{25}(SG)_{18}$ in water.

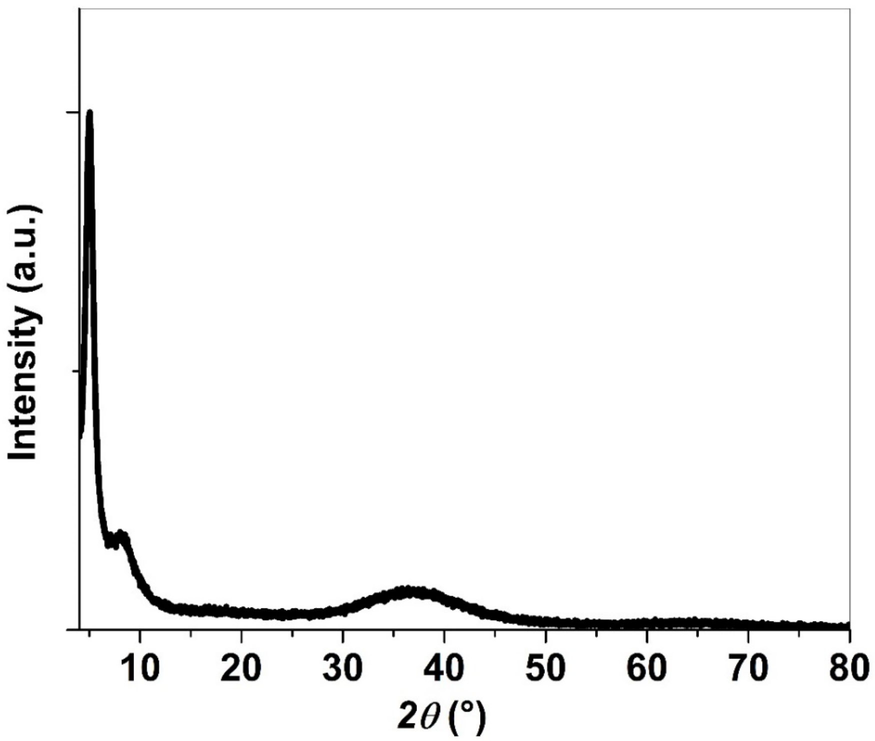

Figure S3: Powder X-ray diffraction pattern of $Au_{25}(SG)_{18}$. The intense reflection at 2θ = 5.01° corresponds to a center-to-center clusters distance of 1.76 nm by applying the Bragg's law [S1].

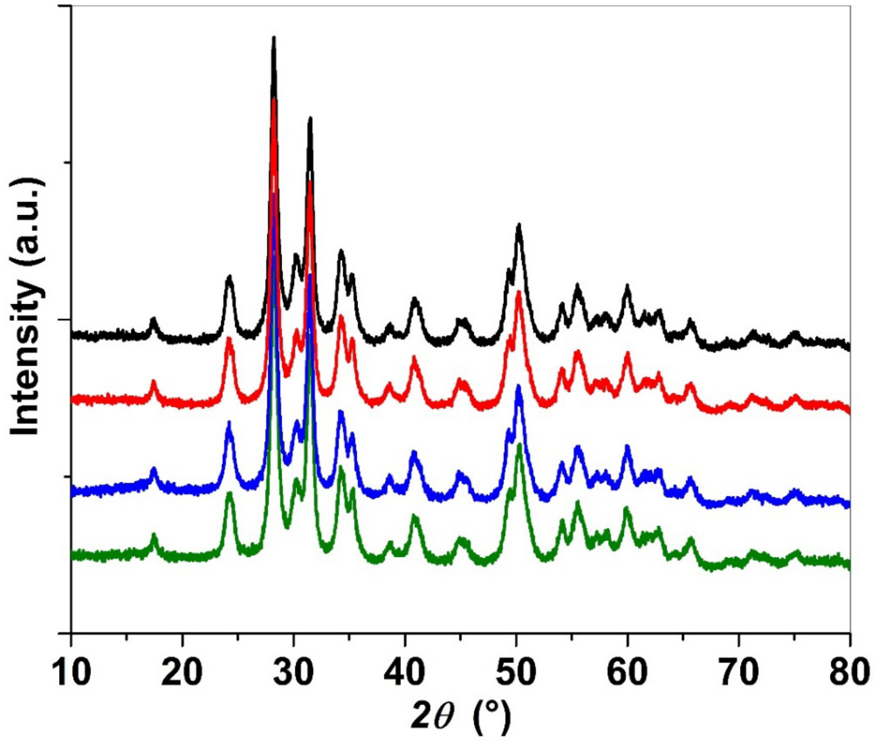

Figure S4: Powder X Ray diffraction patterns of $Au_{25}(SG)_{18}@ZrO_2$ after calcination at 400 ° C for 12 hours under air (red), calcination at 300 °C for 4 hours under air (blue), calcination at 200 °C for 4 hours under air (green), and ZrO_2 support alone (black).

[S1] Lavenn, C.; Albrieux, F.; Bergeret, G.; Chiriac, R.; Delichère, P.; Tuel, A.; Demessence, A. *Nanoscale* **2012,** *4*, 7334.
